# Upper gastrointestinal training in the UK and Ireland: a Roux Group Study

**DOI:** 10.1308/rcsann.2023.0104

**Published:** 2024-04-18

**Authors:** DBT Robinson, R Zakeri, LR Brown, RW Laing, C Choh, A Askari, M Abouelazayem, A Bradley, AC Currie, M Elmasry, RPT Evans, TMH Gall, E Jerome, NB Raftery, M Samuel, HVM Spiers, BKY Chan, ; on behalf of the Roux Group

**Affiliations:** ^1^ The Roux Group, c/o AUGIS, The Royal College of Surgeons of England, UK; ^2^ Institute of Systems, Molecular & Integrative Biology, University of Liverpool, UK

**Keywords:** Education, Upper gastrointestinal surgery

## Abstract

**Introduction:**

Surgical training programmes in the United Kingdom and Ireland (UK&I) are in a state of flux. This study aims to report the contemporary opinions of trainee and consultant surgeons on the current upper gastrointestinal (UGI) training model in the UK&I.

**Methods:**

A questionnaire was developed and distributed via national UGI societies. Questions pertained to demographics, current training evaluation, perceived requirements and availability.

**Results:**

A total of 241 responses were received with representation from all UK&I postgraduate training regions. The biggest discrepancies between rotation demand and national availability related to advanced/therapeutic endoscopy and robotic surgery, with 91.7% of respondents stating they would welcome greater geographical flexibility in training. The median suggested academic targets were 3–5 publications (trainee vs consultant <3 vs 3–5, *p*<0.001); <3 presentations (<3 vs 3–5, *p*=0.002); and 3–5 audits/quality improvement projects (<3 vs 3–5, *p*<0.001). Current operative requirements were considered achievable (87.6%) but inadequate for day one consultant practice (74.7%). Reassuringly, 76.3% deemed there was role for on-the-job operative training following consultant appointment. Proficiency in diagnostic endoscopy was considered a minimum requirement for Certificate of Completion of Training (CCT) yet the majority regarded therapeutic endoscopy competency as non-essential. The median numbers of index UGI operations suggested were comparable with the current curriculum requirements. Post-CCT fellowships were not considered necessary; however, the majority (73.6%) recognised their advantage.

**Conclusions:**

Current CCT requirements are largely consistent with the opinions of the UGI community. Areas for improvement include flexibility in geographical working and increasing national provisions for high-quality endoscopy training.

## Introduction

The year 2021 saw a significant change in the postgraduate surgical training model, from targets driven by indicative numbers to novel curricula across all surgical specialties based on Capabilities in Practice.^1^ This revised structure was deemed to be better aligned with the required skills necessary for day one consultant practice, and offered the potential for acceleration or deceleration of training progression based on acquisition of these skills. Hence, for the first time, this curriculum aims to offer a more tailored approach to training, accounting for the varying speeds by which these targets are met by individuals within the trainee population.

Although many changes have been made to the higher general surgical curriculum, some key similarities to previous iterations remain. Both the indicative operative numbers and the required attainment of three level 4 Procedure Based Assessments (PBAs) in the core emergency surgical procedures, as well as those dedicated to the trainees' chosen individual subspecialty are unchanged. For those pursuing an upper gastrointestinal (UGI) specialty interest, these dedicated procedures include a minimum of 110 cholecystectomies, 35 major UGI procedures (including anti-reflux surgery, bariatric surgery and oesophagogastric [OG] resections or major hepatopancreatobiliary [HPB] procedures) and 200 gastroscopies (except for HPB trainees).^2^

In addition to the operative parameters, all general surgery trainees are required to demonstrate experience and achievements in the fields of academia and research, teaching, and management and leadership. Attendance at a trauma skills course and at national or international conferences is also required. Furthermore, individuals must demonstrate that they have sufficient clinical experience and knowledge, by means of level 4 or level 5 Case-Based Discussion or Clinical Evaluation Exercise, in the curriculum-defined critical conditions and in ten further evaluations based on topics pertinent to their subspecialty interest.^2^

Although the rationale for this new curriculum is based on the changes to clinical practice in the past decade, including a greater emphasis on emergency care, the development of major trauma centres and increased subspecialisation, there is a notable paucity of evidence regarding the experience and expectations of those clinicians who work in the UGI setting.^2^ As such, this study aims to assess the perception of UGI training in the United Kingdom and Ireland (UK&I) from both a trainee and consultant perspective, identify areas for improvement and offer insight into the opinions and suitability regarding the current requirements for the achievement of Certificate of Completion of Training (CCT) in UGI and general surgery.

## Methods

### Questionnaire design and distribution

A cross-sectional questionnaire, tailored to the curriculum, was developed by a consensus group of UK&I trainees with a UGI interest. Results were collected as a combination of binomial and variable scale responses with the option for free text at the end of each section to add any other comments. Questions were broken down into sections to encompass factors relevant to surgical training and trainee outcomes including: demographics, training structure, academic requirements, operative and endoscopic requirements, and fellowship opportunities. The majority of questions were compulsory excluding any follow-on questions based on a previous answer, ‘other comments’ sections and questions where the responses were not relevant to all subspecialty interests within UGI. Google Forms was used to create the questionnaire to enable electronic distribution and to collate the data for analysis. The questionnaire was distributed to both trainee and consultant members of both the Association of Upper Gastrointestinal Surgery and the British Obesity and Metabolic Surgery Society via their mailing lists and at their annual scientific meetings in 2021. To maximise the response rate, the questionnaire was also advertised using the Roux Group's Twitter handle and was open to all postgraduate doctors with a UGI interest in the UK&I. Data collection took place from 21 July to 15 November 2021. A copy of the questionnaire can be found in Figure S1 (available online).

### Statistical analysis

For the purpose of this study, all subconsultant grade surgeons are referred to as trainees. Statistical analysis was performed using SPSS version 27 and R version 4.1.1 with packages including *tidyverse* and *ggplot2*. Categorical data were summarised using frequencies and percentages, and continuous variables were presented using the median and interquartile range. Chi-squared and Mann–Whitney *U* tests were used to compare categorical and continuous data, respectively, and statistical significance was accepted at *p*<0.05.

Regional affiliations were collected as per the UK&I postgraduate regions for training; however, where multiple postgraduate regions were geographically closely affiliated these were combined. Certain questions were only completed if they were relevant to the respondent's subspecialty interest with the aim of limiting any bias that may skew the data. As such, not all respondents answered these questions. For questions regarding whether the current structure of training allows sufficient experience in each of the UGI subspecialties, those who answered ‘Don’t know’ were similarly excluded from the analysis.

### Ethical approval

The Health Research Authority decision tool was used and determined that National Health Service Research Ethics Committees review was not required. Ethical approval was deemed not to be required because the work represents service evaluation. Completion of the questionnaire was taken as implied consent to participate in this study.

## Results

### Demographics

**Figure 1:**
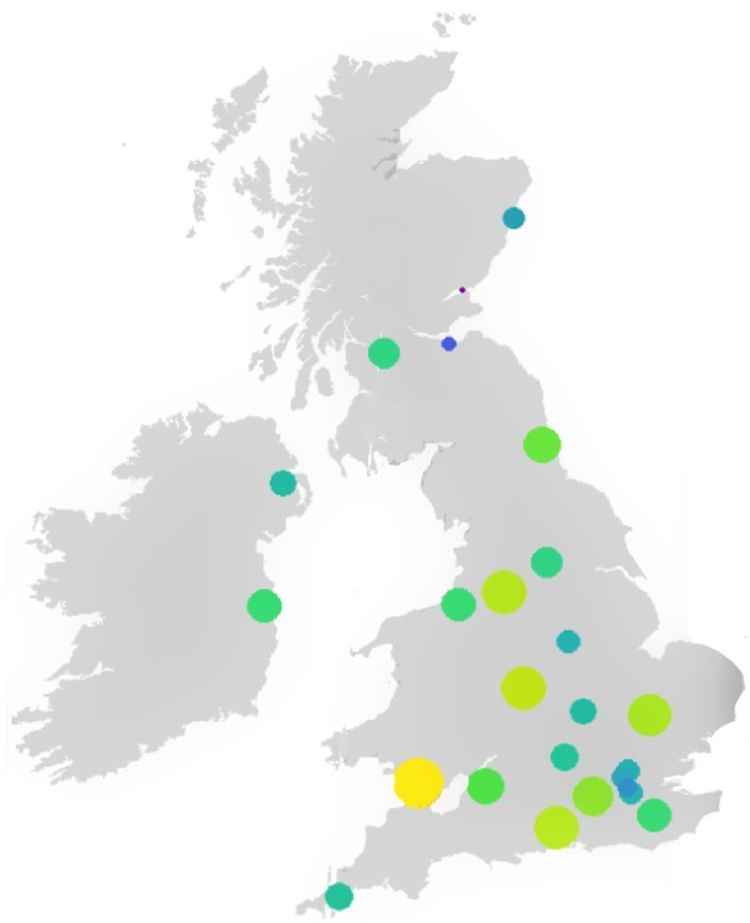
Responses by postgraduate training region. Bubble chart with larger and lighter dots representing more respondents.

**Figure 2:**
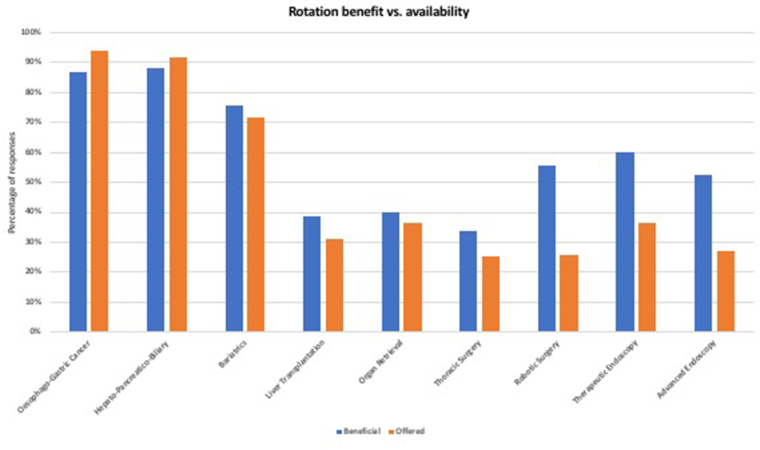
Rotation benefit vs availability. HPB = hepatopancreatobiliary; OG = oesophagogastric.

Two hundred and forty-one individuals (74.3% male, 24.5% female, 1.2% preferred not to say) completed the questionnaire. Responses were received from all postgraduate training regions across the UK&I with responses by region ranging from 6 (Northern Ireland) to 26 (Wales) (Figure 1). The majority of respondents undertook their primary medical degree in the UK&I (72.2%) with an even greater proportion of respondents pursuing/having pursued their postgraduate training in the UK&I (87.1%). Although respondents represented all stages of postgraduate surgical training, the largest group were surgeons in training of ST3–ST8 (49.4%). Overall, trainees accounted for 59.8% of all responses, with consultants making up the remaining 40.2%. All subspecialty interests were represented, with resectional OG surgery being the largest group (31.1%) and liver transplantation (2.1%) the least predominant (Figure 2). Nineteen (7.9%) individuals were undecided regarding an UGI subspecialty interest.

### Length of training

The median/anticipated time from completion of primary medical degree to CCT was 13 years and the median/anticipated age at that point was 35–39 years old, with 65.5% of respondents falling into this category. There were no differences between trainees and consultants regarding the time to or age at CCT. Overall, length of training was regarded as reasonable (reasonable [50.2%] vs too long [40.7%] vs too short [9.1%]). Yet, subanalysis revealed that views differed between consultant (reasonable [61.9%] vs too long [21.6%] vs too short [16.5%]) and trainee respondents (reasonable [42.4%] vs too long [53.5%] vs too short [4.2%]; chi-squared 28.5, *p*<0.001). With regards to the structure of training, 67.2% felt that it was unfocused, driven more so by trainee rather than consultant opinion (79.9% vs 48.5%; chi-squared 25.9, *p*<0.001). Despite this only 24.5% felt that it was incomplete and in contrast to the subject of focus, this view was shared by trainees and consultants alike (28.5% vs 18.6%; chi-squared 3.1, *p*=0.079).

### Variations in training preference and availability

**Figure 3:**
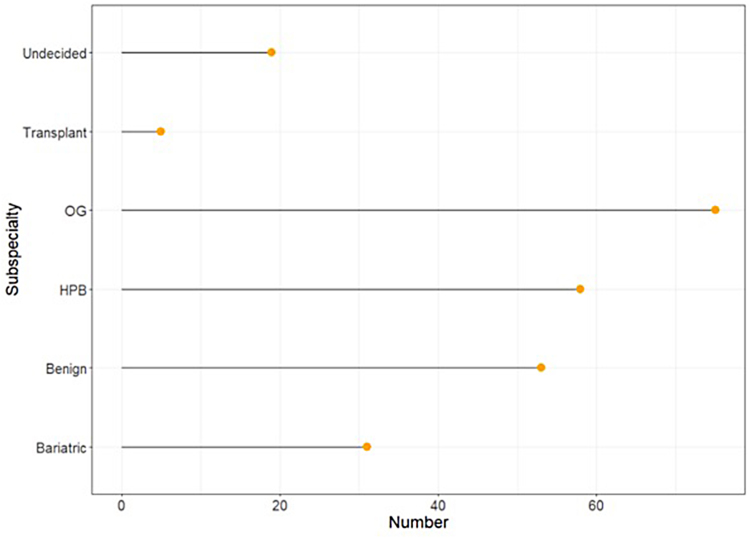
Subspecialty variation among respondents. HPB = hepatopancreatobiliary; OG = oesophagogastric.

Figure 3 highlights the variations in the perception of subspecialty rotation benefit vs the access to such rotations as described by respondents. Not all deaneries offered all the listed options of training rotations. The potential to offer trainees opportunities to travel to neighbouring deaneries to gain experience in areas that were not locally available was overwhelmingly positive, with 91.7% of respondents believing that this should be encouraged. Moreover, this opinion was shared by both the trainee and consultant cohorts (chi-squared 0.6, *p*=0.439). Only one (0.4%) individual felt that this should not be the case; a further 19 (7.9%) individuals were uncertain.

### Academic requirements for CCT

**Figure 4:**
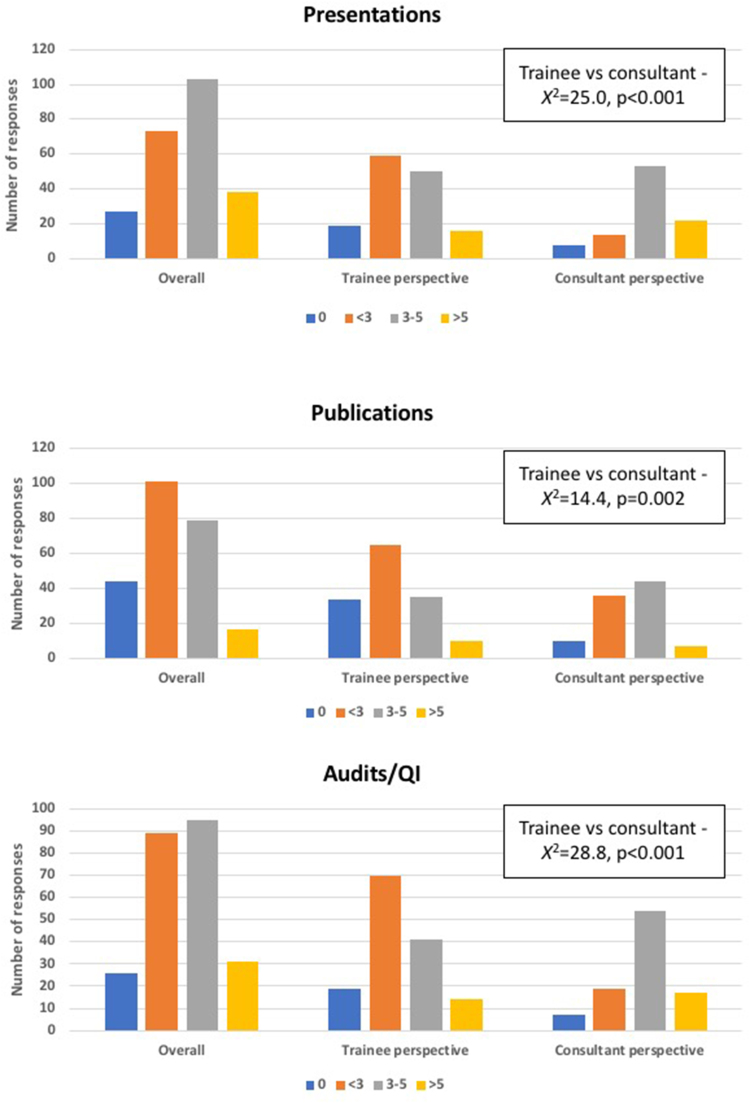
Overall, trainee and consultant perspectives regarding appropriate academic requirements for Certificate of Completion of Training (CCT)

The academic requirements for CCT encompass presentations, publications and completion of audits or quality improvement (QI) projects. The median suggested targets for these, as defined by the respondents, were 3–5, <3 and 3–5, respectively. Further variation was seen between the trainee and consultant cohorts, with most consultants suggesting targets of three to five for all three academic components, whereas trainees felt that fewer than three of each would be more appropriate (Figure 4). Nonetheless, the majority of both cohorts recognised the need for some academic requirements with the minority across both groups selecting none for each of the academic criteria.

Although the study's respondents appreciated the benefits of a dedicated period of out-of-programme research (OOPR), the general consensus is that a higher degree should not be a prerequisite to CCT even though it was considered advantageous for future career progression (required [9.7%] vs not required [28.4%] vs advantageous but not compulsory [61.9%]). Despite an overall minority of both the trainee and consultant cohorts believing that a higher degree should not be a CCT requirement, consultants were significantly more likely to believe it should be (15.8% vs 5.7%; chi-squared 8.2, *p*=0.017). Regardless, the majority (63.5%) of respondents either had or planned to take a period of OOPR, with trainees and consultants similar in this regard (58.3% vs 71.1%; chi-squared 5.8, *p*=0.055). The most common reason for taking OOPR time was an academic or subject interest (51.7%) followed by an intention to make oneself more competitive for consultant positions (35.5%) and a desire to pursue an academic career (23.5%). Figure S2 (available online) demonstrates the responses received regarding OOPR.

### Endoscopic requirements for CCT

The majority of respondents feel that proficiency in diagnostic endoscopy should be a minimum requirement for CCT and consultant practice (81.7% and 67.6% respectively). Therapeutic endoscopy, endoscopic retrograde cholangiopancreatography, endoscopic ultrasound and endoscopic mucosal resection competence by CCT were considered essential by 33.2%, 3.3%, 1.2% and 1.4%, respectively, with no significant differences between the views of trainees or consultants (all *p*>0.05). By contrast, the majority of respondents felt that therapeutic endoscopy was a necessary skill for consultant practice (53.1%). Similarly, a greater proportion of individuals felt that endoscopic retrograde cholangiopancreatography, endoscopic ultrasound and endoscopic mucosal resection were similarly essential for consultant practice (9.1%, 10.8% and 11.2%, respectively); however, this still represented a minority and the increase was primarily driven by trainee responses (all *p*<0.05), unlike the increase seen with therapeutic endoscopy which was agreed by both cohorts (*p*=0.689).

### Operative requirements for CCT

The majority (87.6%) of respondents felt that the current CCT operative requirements are achievable. However, only 22.8% perceived them to be sufficiently specific, with 74.7% stating that the requirements are inadequate for day one consultant practice. This opinion was shared by both the trainee and consultant groups (75.7% vs 73.2%; chi-squared 0.2, *p*=0.662). It was uniformly (*p*>0.05) felt that the current national structure does not allow sufficient experience in resectional HPB, resectional OG, bariatric or liver transplantation surgery. Consequently, combination training such as resectional HPB and transplant or resectional OG and bariatric surgery were deemed to be even less achievable. Reassuringly, 76.3% deemed there to be a role for on-the-job formalised mentorship operative training following consultant appointment, with both cohorts agreeing this on subanalysis (86.8% vs 89.5%; chi-squared 0.3, *p*=0.559).

**Figure 5:**
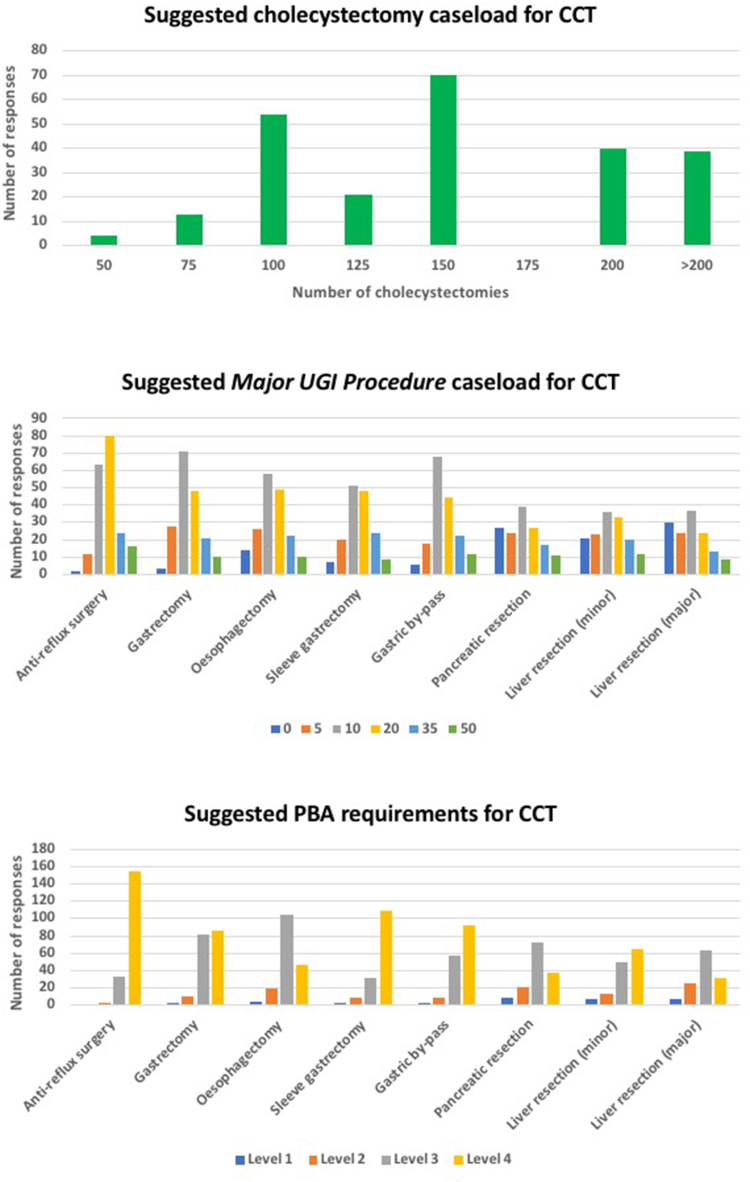
Suggested operative requirements for Certificate of Completion of Training (CCT). PBA = Procedure Based Assessment; UGI = upper gastrointestinal.

The median number of cholecystectomies suggested for CCT was 150. For major UGI procedures 20 anti-reflux procedures and sleeve gastrectomies were deemed appropriate, whereas a median of 10 gastrectomies, oesophagectomies, gastric bypass procedures, pancreatic resections and minor and major (3+ segment) liver resections each were suggested to be suitable targets (dependant on the individuals UGI subspecialty of choice). Furthermore, level 4 was the median PBA deemed necessary in anti-reflux procedures, sleeve gastrectomy and gastric bypass by CCT, with level 3 PBA for all other procedures being suggested as an appropriate target (Figure 5). There were no differences in the suggested indicative numbers or PBA targets between the trainee and consultant cohorts (all *p*>0.05).

### Post-CCT fellowship

Despite the aforementioned results, only 17.4% of individuals felt that a fellowship was necessary for consultant appointment; however, the majority (73.4%) recognised it to be advantageous (but not necessary). The perspective varied between trainee and consultant respondents, with significantly more consultants feeling that post-CCT fellowship is necessary (11.8% vs 25.8%; chi-squared 7.9, *p*=0.020).

For those trainees who wish to undertake a fellowship, the median minimum number of each specialist procedure to aspire towards was universally 20, with 50% of these cases being minimally invasive. Regardless of the subspecialty interest, following a 12-month fellowship there was a consensus that trainees should be proficient (level 4 PBA) in the advanced procedures pertinent to their future consultant practice. Again, there were no differences in the suggested indicative numbers or PBA targets between the trainee and consultant cohorts related to 12-month fellowship posts.

### Gender variations

Table S1 one shows a comparison of responses related to gender. No statistically significant variations were identified across any of the question domains asked with the exception of career stage and subspecialty interest. There were significantly more female trainees than consultants (trainee vs consultant, 81.4% vs 18.6%) compared with the male cohort (trainee vs consultant, 53.1% vs 46.9%; *p*<0.001), with females showing a greater interest in benign UGI (27.1%) and males preferencing OG surgery (33.5%).

## Discussion

This is the first study to gain a national perspective regarding the current UGI curriculum in the UK&I. The principal findings of this study showed that the current CCT requirements are largely consistent with the opinions of the UGI community as a whole. Areas for development include increased flexibility in geographical working, allowing trainees opportunities to develop skills not locally available, and greater access to endoscopy training for UGI surgeons. Generally, consensus was achieved between trainees and consultants with the biggest differences in opinion relating to academic performance, with consultants having greater expectations, and the necessity of OOPR and post-CCT fellowships, where consultants were more likely to deem these necessary rather than just advantageous. There were no significant gender-related variations outside the demographic data.

Trainees consider many factors when choosing a postgraduate training region. Commonly, these include personal rather than professional considerations such as distance from the family home, a preference to stay close to the location of their undergraduate degree or the attraction of metropolitan cities.^3–5^ However, the availability of specific subspecialists opportunities is less likely to be a consideration earlier on in an individual's career, such as at the time of applying to core or higher surgical training, but clearly has important ramifications later on. This study has demonstrated a lack of equality in training with significant geographical variation in training rotation availability, and although it is currently possible to undertake a period of training in another postgraduate region, unlike dedicated periods of research, there is no set pathway or protocol for this. Moreover, the feasibility of a period of training in another postgraduate region is dependent on the authorisation of both the individual's training programme director and their existing school of surgery, which naturally contributes to the inequality. Based on the findings of this study, measures to facilitate interregion relationships including the introduction of a formalised protocol to standardise this approach would be welcomed.

Access to endoscopy training in the UK&I is known to be discrepant, with multiple studies suggesting poorer access to dedicated weekly endoscopy training lists for surgeons when compared with their medical counterparts. A survey conducted by Ratcliffe *et al* in 2022 identified that only 39.8% of surgical trainee respondents had access to a weekly training list compared with 70% of gastroenterology trainees.^6^ Equally, Patel *et al* in 2019, examined the barriers to training in endoscopy among gastrointestinal surgeons and reported that only 28.6% of their study’s respondents were timetabled weekly endoscopy training lists, with 28.1% having no training opportunities at all. Barriers to training included a lack of available lists (77.2%), conflicting operative commitments (59.4%), preferential allocation to gastroenterology trainees (57.9%) and resistance from endoscopy departmental leads (38.6%).^7^ These findings, coupled with the resulting backlog secondary to the COVID-19 pandemic and the fact that over 50% of gastroenterologists are predicted to retire in the next decade, suggest that there is an endoscopy crisis brewing.^8^ The latest iteration of the general surgery curriculum has seen gastroscopy mandated for UGI trainees to attain their CCT and this study has demonstrated not only an expectation, but also an appetite for diagnostic and therapeutic endoscopy training among the UGI community.^2^ Improving surgical trainees' access to endoscopy training may offer, in part, a solution to this pending crisis.

The opinions of trainee and consultant respondents were grossly in agreement across the spectrum of questions asked. However, across the academic requirements for CCT (presentations, publications and audits/QI projects), consultants had greater expectations than the trainee cohort. The previous iteration (2013) of the general surgery curriculum set a minimum requirement of three presentations, three publications and three audits/QI projects, which aligns with the targets suggested by the consultant cohort in this study.^9,10^ The current curriculum (2021) sets no minimum number for presentations or publications, but maintains the requirement of three audit or QI projects. Despite the reduced emphasis on research activity, the curriculum does still maintain that an ability to practise evidence-based medicine, critically appraise literature, apply basic research principles and show an understanding of research governance must be demonstrated.^2,11^ It could, therefore, be argued that the easiest way to do this would be to publish and present research. As such, the suggested targets of fewer than three, but importantly some presentations, publications and audits/QI projects, as expressed by the trainee cohort, aligns more closely with the new curriculum. Ultimately, although the suggested targets of trainees and consultants are different, there is a general consensus that scholarly activity in all three domains is integral to surgical practice and should therefore comprise a component of the curriculum.

Academic targets have been shown to be facilitated by dedicated OOPR time.^12,13^ Yet, while the majority of respondents considered a higher degree advantageous for future career progression, fewer than 10% felt that this should be a requirement, despite two-thirds stating that they had or were planning to undertake an OOPR. Although the majority of both cohorts agreed, the consultant cohort were significantly more likely to describe it as a necessity. A similar picture was seen with regards to post-CCT fellowships. Despite the general consensus that the skills required for consultant subspecialist practice are difficult to achieve in the current model of training before CCT and an expectation that those wishing to pursue bariatric or resectional OG/HPB surgical careers will not be competent to independently perform the role by this stage, only a minority felt that a fellowship was necessary. This is even more surprising given that the cohort as a whole agreed that the current operative requirements are achievable but not adequate for independent day one consultant practice. Like OOPR, consultants were significantly more likely to describe post-CCT fellowship as necessary, with more than one-quarter of the cohort stating so. There are few data in the literature regarding whether or not a higher degree or a post-CCT fellowship truly increases your likelihood of a consultant appointment. Yet, it could be argued that these additional qualifications/experiences have become a surrogate way in which to differentiate between candidates at consultant appointment owing to the bottleneck for resectional UGI positions, with most OG/HPB resectional surgeons undertaking both a higher degree and fellowship, but disappointingly few consultant jobs offer the time or funding opportunities for continuing research activity. Although some reassurance may be taken from the overwhelming perception that there is a role for on-the-job operative mentorship training following consultant appointment, trainees should not ignore the opinions of their consultant counterparts or the limitations of the current training programme, as outlined in this study, when making their decision as to whether to undertake such opportunities.

The intention of the new curriculum is to align more closely with achieving the skills needed for day one consultant practice. Nevertheless, the length and focus of training to achieve consultant status within UGI surgery have been found wanting. In 2020, Whewell *et al* examined the variations in training requirements within general surgery across 23 countries and reported that the number of years in postgraduate training ranged from 4 to 10, with the UK reported as having the longest postgraduate course, closely followed by Ireland (9 years).^14^ The national working hours restrictions for each country were also explored. Greece, Ireland, Italy, The Netherlands, Switzerland and the UK are all governed by the European Working Time Directive, which limits the average working week to 48 hours. Despite this regulation being applicable in each of these countries, with the exception of the UK and Ireland, the duration of postgraduate training was 6 years, demonstrating that with greater refinement and focus postgraduate surgical training is feasible in a shorter time frame.

### Study limitations

Limitations include the survey methodology mapped specifically to the current CCT guidelines and quantitative results presented. Given that only a proportion of trainees undertaking general surgical training become UGI trainees, it is not surprising that the survey had a small number of respondents. It did have a good spread of demographics, making it representative.

## Conclusion

In conclusion, the current CCT requirements as outlined by the Joint Committee on Surgical Training are consistent with the opinions of the UGI community, with trainee and consultant respondents largely in agreement. Targeted areas for improving UGI training in the UK&I include increasing geographical flexibility to enable equitable access to all elements of UGI surgery and ensuring adequate provisions nationally for high-quality endoscopy training. Refinement of the current training programme would be advantageous to aid acceleration of the acquisition of the skills needed for consultant practice to enable the UK&I to align more closely with other global healthcare settings.

## Data availability

All data pertaining to this study are available upon reasonable request to the corresponding author. Questionnaire responses have been anonymised and thus the risk of respondent identification is low.

## Funding

BKYC is supported by the North West England MRC Fellowship Scheme in Clinical Pharmacology and Therapeutics, which is funded by the MRC, Eli Lilly and Co., Novartis, Roche Pharma, UCB Pharma, the University of Liverpool, and the University of Manchester (MR/N025989/1).

## Authors’ contributors

DBTR: concept and design of the work; acquisition, analysis and interpretation of data for the work, and writing of the first draft. RZ: Design of the work and acquisition of data; critically appraised and suggested revisions to first draft in the development of the submitted draft. LB: Design of the work and analysis and interpretation of data; critically appraised and suggested revisions to first draft in the development of the submitted draft. RWL: Concept and design of the work and acquisition of data; critically appraised and suggested revisions to first draft in the development of the submitted draft. CC: Design of the work and acquisition of data; critically appraised and suggested revisions to first draft in the development of the submitted draft. AA: Design of the work and acquisition of data; critically appraised and suggested revisions to first draft in the development of the submitted draft. MA: Design of the work and acquisition of data; critically appraised and suggested revisions to first draft in the development of the submitted draft. AB: Design of the work and acquisition of data; critically appraised and suggested revisions to first draft in the development of the submitted draft. AC: Design of the work and interpretation of the data; critically appraised and suggested revisions to first draft in the development of the submitted draft. ME: Design of the work and acquisition of data; critically appraised and suggested revisions to first draft in the development of the submitted draft. RPTE: Design of the work, acquisition and interpretation of data; critically appraised and suggested revisions to first draft in the development of the submitted draft. TG: Design of the work and acquisition of data; critically appraised and suggested revisions to first draft in the development of the submitted draft. EJ: Design of the work; critically appraised and suggested revisions to first draft in the development of the submitted draft. NR: Design of the work; critically appraised and suggested revisions to first draft in the development of the submitted draft. MS: Design of the work and interpretation of the data; critically appraised and suggested revisions to first draft in the development of the submitted draft. HVMS: Design of the work and acquisition of data; critically appraised and suggested revisions to first draft in the development of the submitted draft. BKYC: Concept and design of the work; acquisition, analysis, and interpretation of data for the work, writing of first draft. All authors have given final approval of the version being submitted and agree to be accountable for all aspects of the work.
